# From the field: The importance of ophthalmic nurses: an ophthalmologist's view

**Published:** 2020-12-31

**Authors:** Michelle Hennelly

**Affiliations:** 1 MSc Programme Director in Clinical Optometry: Division of Optometry and Visual Science, City, University of London, UK.

**Ophthalmologist Hillary Rono spoke with our consulting editor for this issue, Michelle Hennelly, about the importance of ophthalmic nurses in bringing eye care to the community.**

**Figure:**
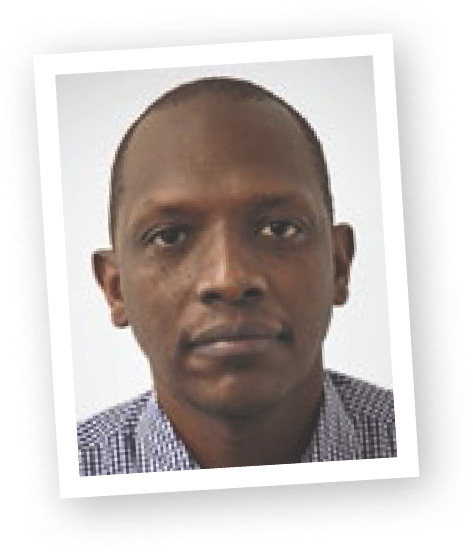


*After completing his Master of Medicine Degree in Ophthalmology, Hillary Rono chose to be posted to an area in the North Rift region in Kenya, where he coordinates outreach programmes to remote areas.*

## How have ophthalmic nurses helped you as an ophthalmologist?

In my role as a clinician, ophthalmic nurses help me to triage patients (filter the urgent from the non-urgent), administer eye drops, and - during outreach - identify patients that need to be treated in hospital. They are also very good managers of eye care programmes and link very well with other parts of the hospital, such as maternal and child health service.

## How can ophthalmic nurses contribute to research in Kenya?

Ophthalmic nurses are key people in terms of supporting eye care research. Already, they have the skills to identify symptoms and understand the complexities of eye care. They assist in theatre and are critical in infection control measures. They are able to educate patients and teach other eye care workers too, thereby passing eye care skills to other health care workers.

## Training of nurses and support staff is essential. What has worked so far, and how can training in the region be enhanced?

After I arrived in 2006, we set up a short 3-month skills upgrade course (the ‘Ophthalmic Assistant’ course) that taught general nurses in the area how to manage simple eye diseases and how to identify cataract and eye emergencies. The course introduced more nurses to eye health as a potential specialty and made it possible for us to identify and encourage those with a strong interest in eye health to complete a one-year postgraduate diploma in ophthalmic nursing. This programme helped us to cover the shortfall in the area, and it was later adopted nationally.

Looking ahead, improving the eye care skills of general nurses would be very useful, especially in areas where there are no optometrists. Unfortunately, ophthalmology is given the least priority in basic general training. For example, the general nursing diploma course, which takes three years, includes only 4-5 hours of ophthalmology; this does not give nurses enough exposure.

**Figure:**
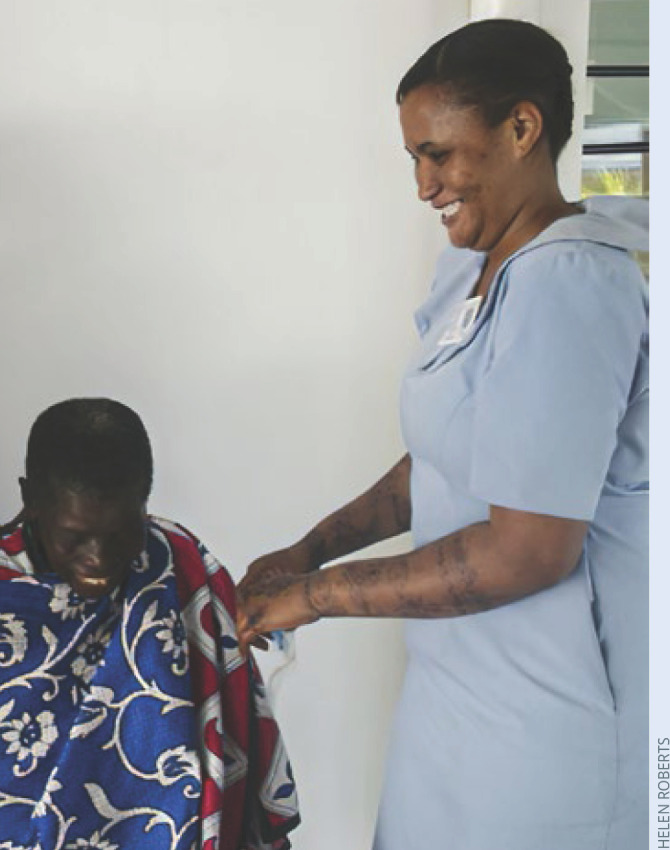
Ophthalmic nurses are vital members of the eye team in Kenya.

## What are some of the unique ways in which ophthalmic nurses contribute to eye care?

Ophthalmic nurses don't often use the clinical jargon that many ophthalmologists use, so they are more successful in getting important messages across with regards to patients' management of their own eye care.

There is something unique about the relationship between ophthalmic nurses and patients, which could be due to their nursing training; however, this could also be because they spend more time with patients compared to ophthalmologists. Ophthalmic nurses can also speak up on behalf of the patient, which builds trust. This makes ophthalmic nurses excellent at obtaining good clinical histories.

Patients can be very nervous at the start of treatment or a procedure, but after speaking to an ophthalmic nurse they trust they can feel reassured and secure in the care that they are going to receive.

## What are the challenges involved in retaining ophthalmic nurses?

Ophthalmic nurses are general nurses first, so they can often be called upon to help in areas where they worked previously, such as labour wards or in child health, when these are short-staffed. It is very important that eye unit managers lobby the hospital management to explain the specialist role and skills of these nurses so that their importance can be recognised and they can be retained in their posts.

